# A Comprehensive Mixed Methods Approach for Studying the Quality of Life of Colombian Periodontists

**DOI:** 10.3390/ijerph192316102

**Published:** 2022-12-01

**Authors:** Wilmer A. Romo-Cabrera, Andrés A. Agudelo-Suárez

**Affiliations:** Faculty of Dentistry, University of Antioquia, Medellín 050010, Colombia

**Keywords:** mental health, periodontists, quality of life, qualitative research, research technics

## Abstract

This study analyzed the association of the sociodemographic, labor, and health conditions on the quality of life (QOL) of the periodontists in Colombia. A mixed study (explanatory sequential design) was conducted. The first quantitative phase was carried out by a cross-sectional survey (*n* = 187; 60.4% females). Variables: sociodemographics, labor, and health conditions, QOL (WHOQOL-BREF). Normality tests, descriptive statistics, and bivariate analyzes were performed. Factors associated with QOL were established using multivariate linear regression. A second qualitative phase with two focus groups (FGs) delved into those aspects of relevance, through qualitative content analysis and triangulation of information. The multivariate linear regression analysis showed that the factors associated with the QOL scores were in the case of men and negatively (QOL decreases): having low social support (*p* < 0.001), reporting poor mental health (*p* < 0.01). For women, QOL scores were negatively associated with low social support (*p* < 0.001), reporting poor general and mental health (*p* < 0.01), having greater stress at work (*p* < 0.05), having a temporary contract (*p* < 0.05), and living in a medium or low socioeconomic status (*p* < 0.05). The analysis of the FG allowed us to understand how QOL is permeated by the social context. The specialization of periodontics has generated a change in living conditions, and an adaptation to the workplace that allows them to gain recognition and a higher QOL. In conclusion, the QOL of periodontists is defined in subjective terms (standards) and related to social and labor conditions. Follow-up and evaluation strategies of the general conditions of these clinical specialists in Colombia are required.

## 1. Introduction

Quality of life (QOL) has been defined from numerous approaches and perspectives and denotes, in general terms, a multifactorial and multidimensional concept [1]. Although this concept initially refers to a significant element based on the individual and personal experience, there are social determinants that are closely linked to the perception of good or bad QOL in different scenarios [1,2,3]. QOL is related to biological and functional concepts, such as health status, and social and psychological concepts such as well-being, satisfaction, and happiness, and there is even talk of spiritual aspects [3,4]. This QOL can be differential between population groups and one of them that is important and sensitive to be affected in terms of their QOL is the health personnel and especially the group of dentists [5,6,7].

From this perspective, a linked element to QOL is the labor aspect. In fact, there exists some research focused on evaluating the QOL at work and/or labor well-being [8]. For instance, it has been found that job satisfaction for healthcare workers is declining [9], while there is moderate/high satisfaction in dentists, even though specialists were more satisfied than general dentists [10]. The factors that positively influenced QOL were the relationship with the patient, respect, dental staff, professional relationship, and the workplace; and those that negatively influenced included personal time, stress, income, practice management, and professional experience [5,11].

For dentists, including their specialties, it has been observed that QOL is a matter of growing concern [7]. This situation can be attributed to the fact that dentists need to exert great physical and mental effort to keep up with the requirements of patients who expect precise and efficient treatments, which needs to be supported by constant updating of knowledge and technology [5]. Similarly, QOL could be influenced by political aspects.

For instance, the neoliberal economic model has generated some changes with regard to professional work of the health workers and specifically on the general and specialized dentistry [12]. Previous research has shown an impoverishment in employment and working conditions, a greater demand and labor mobility with the consequent extension of working hours, and several inter-municipal jobs to gather enough income to get closer to economic expectations [12,13]. These aspects could affect the professionals’ QOL and subsequently their ability to provide high-quality dental care [13,14].

Studies conducted on QOL within oral health professionals use instruments that allow its assessment through the individual experience including multiple factors and spheres of human, social, and work development [5,6,7,14]. From the above discussion, more studies in Colombia are compulsory, which recognize the particularities of the different dental specialties and the context in which they operate. That is the reason why different methodological approaches are required and, in particular, mixed studies offer an engaging field of action in the scientific agenda, addressing complex realities from various analysis instruments and allowing comprehensiveness in their interpretation [15]. This study assumes as a hypothesis or theoretical assumption that the QOL is permeated by individual and contextual conditions, as well as the particular sociodemographic and labor conditions of periodontists in the country. The collected information allows the implementation of monitoring strategies for the general conditions of the dental specialties in the country. Specifically, this study analyzed the association of the sociodemographic, labor, and health conditions on the quality of life (QOL) of the periodontists in Colombia.

## 2. Materials and Methods

### 2.1. Design

A mixed-methods study with an explanatory sequential design was proposed [15,16]. In this type of study, the qualitative component helps in interpreting quantitative findings. This methodology allows to explain a phenomenon, interpret unexpected results, or explore some results in greater detail (in this case, the QOL and the related factors) [15,16]. A similar methodology as in previously published studies was used [14,17], using other context-specific variables for periodontists. The protocol of this research was not preregistered.

### 2.2. Quantitative Sub-Study (Cross-Sectional)

A cross-sectional online survey (by using the Google Forms tool) was applied. As participation criteria, those dentists residing in Colombia, with a specialist title in periodontics or related areas, who also carried out their professional practice in the country and who wished to participate, were included. Other specialists and professionals who were unable to be contacted or accepted to participate in the study were not included. Various strategies to recruit people were used: emails provided by associations of periodontists, use of social networks (Facebook and WhatsApp), nominal sampling or snowballing through referrals, and forwarding the questionnaire to colleagues and relatives. The fieldwork of the survey was conducted between Abril-October 2021 and a final sample of 187 participants (113 women; 60.4%) was achieved. A pilot study was carried out on a sample of 10 periodontists to improve the intelligibility of the questionnaire, and to assess the time to completion for the respondents and the internal consistency of the questions.

Quality of life (QOL) was measured by the WHOQOL-BREF [18,19]. This questionnaire comprises 26 items distributed on four dimensions: physical, psychological, social relationships, and environment. We followed the guidelines proposed by the study group by establishing the scores for each dimension (higher scores indicate higher QOL levels). Other variables that were included are: sociodemographic, employment conditions, sport practice, self-rated general health, self-perceived stress, mental health (GHQ-12) [20,21], functional social support questionnaire (Duke-UNC-11) [22,23].

Reliability testing was performed by calculating the Cronbach’s alpha coefficient for WHOQOL-BREF, GHQ-12, and Duke-UNC-11 instruments in the sample. The following values were obtained: (1) WHOQOL-BREF: Global: 0.919 and for the domains physical: 0.766; psychological: 0.800; social relationships: 0.742; environment: 0.845; (2) GHQ-12: 0.710; (3) Duke-UNC-11: 0.902 (acceptable in all cases for the study purposes).

SPSS software version 22.0-IBM^®^ for Windows (Armonk, NY, USA: IBM Corp.) was used to carry out all of the analyses (separately for men and women). All variables underwent descriptive analysis. Normality distribution was tested by the WHOQOL-BREF and other quantitative variables (Kolmogorov–Smirnov test). Tests of statistical significance were carried out to observe differences among variables according to their nature (Mann–Whitney U test for dichotomous variables, Kruskal–Wallis test for polychotomous variables, and the Spearman correlation for quantitative variables).

A linear multivariate regression analysis was carried out to identify possible predictors of the scores of QOL by using the stepwise method. Four multivariate linear regressions (for the same quantity of domains of the WHOQOL-BREF) were calculated including sociodemographic (*n* = 6), labor (*n* = 22), and health (*n* = 4) variables. Belonging was determined by evaluating the compliance with the assumptions of linearity, non-collinearity and normality, constant variance, and correlation of residuals. Due to the specificities of the statistical calculations in the software, different models (adjusted and unadjusted) were carried out. Non-statistically significant variables were not included in the definitive adjusted models. For the physical domain, seven models were made for females and two models for males where the adjusted final model only included seven variables with statistical significance for women and two variables for men. For the psychological domain, two models were performed for women and three for men and the final adjusted model included two statistically significant variables for women and three variables for men. In the case of the social relationships domain, two models were carried out for women and three models for men, and the adjusted final model included two and three statistically significant variables for men and women respectively. Finally, for the environmental domain, two models were carried out for women and a single model was established for men. The adjusted final model included two statistically significant variables for females and one for males.

### 2.3. Qualitative Sub-Study (Focused Ethnographic Perspective)

Two focus groups (FGs) were carried out and participated teen respondents that previously completed the online survey (selected for convenience). This technique was chosen to obtain a collective discourse by the dialogue and interaction of the participants. FGs were conducted by the research team using Microsoft Teams (remote means). Through the opinions and experiences of the participants, we dept on the information obtained from the survey. For that purpose, the research group designed a guide of topic to be included in the FGs and we focused on the factors that determine the quality of life in this group, by analyzing their particular context. We asked for the concept of quality of life, social and particular determinants, the general context of periodontitis, and variables significantly associated with QOL. FGs lasted between 90 and 120 min and were digitally recorded and transcribed verbatim. Fieldwork of FGs was carried out between March and April 2022. A thematic analysis was carried out to recognize trends of information found in the participants’ discourses. All analyses were carried out manually (without using support software). The text fragments were grouped into four categories.

### 2.4. The Methods Integration Approach

Triangulation methods were applied, achieving the integration of both substudies [15]. An explicative conceptual map was formulated, according to individuals’ opinions of this specialists’ group.

### 2.5. Ethics

The research process followed Colombian regulations (Resolution No. 008430/1993- Ministry of Health and Social Protection) and the bioethics research committee of the Faculty of Dentistry of the University of Antioquia (Act 06/2020, Concept No. 54) approved this study. Individuals’ participation was voluntary and informed consent was obtained from study participants.

## 3. Results

### 3.1. Quantitative Aspects

#### 3.1.1. General Profile of the Participants of the Study

Table S1 (Supplementary Materials) shows the sociodemographic, labor, and health characteristics of the study participants. The median age was 43 ± Interquartile Range -IQR- 13 years for males (a little less for women). Nearly two-thirds of the men were married. Almost half of the participants lived-in high-status places, and most had a vehicle. There is a slightly higher percentage of men who live alone than women. More than 60% live in their own homes. The median years of experience as a periodontist range between 9 and 10 years. Most work in clinical activities. A large section does not have written contracts, and very few expressed that they have permanent contracts.

The working day had a median of between 35 and 40 h/week, and 19% of women and 58% of men earn more than 6 million Colombian pesos per month (approx. 1500 U$). Between 72 and 92% of those surveyed consider the salary to be enough to cover different types of expenses, although 40% of women and 55% of men mention not being well paid for the time they dedicate to work. Moreover, 30% of women mention being dissatisfied with their job and 84% of men mention that the job is stressful. There is evidence of a median annual participation of 2 ± IQR 2 annual academic events in men.

Considering the health variables, 74% of males and 43% of females mentioned sports practice. 85% and more of both sexes referred to having a good self-rated health, although 62% of males and females perceived poor mental health. A low social support is perceived in 13% of women (a lower percentage in men). Considering the WHOQOL-BREF domains, the median of scores surpassed 59 points, being higher in the physical dimension and lower in the environment. In almost all cases, the QOL scores are lower in women, with the exception of the social relationships domain (the score is the same).

#### 3.1.2. Variables Related to QOL and Its Different Dimensions

In Table S2 (Supplementary Materials), the bivariate correlations between the QOL domains and the quantitative variables are shown. In case of women, statistically significant and positive correlations were found according to the environmental dimension and the variables age, experience as a dentist/periodontist, and the physical dimension with the number of hours of work per week. In case of men, there were statistically significant and positive correlations between the social dimension and the years of experience as a periodontist and the number of hours of work per week and the environment dimension with the annual frequency of academic events.

Table 1 presents the bivariate comparisons of the dimensions of the WHOQOL-BREF instrument for QOL and the qualitative variables of the study according to sex. In case of women, there were statistically significant differences for the socioeconomic stratum variables and the dimensions analyzed except for the physical domain (>score in high socioeconomic status). Having a vehicle is significantly related to the psychological dimensions and the environment. Having a percentage rent contract is significantly and negatively related to the environmental domain while having a permanent contract is positively associated with the physical, psychological, and environmental dimensions. Earning more than 5 million Colombian pesos (approx. US$1250 at the time of the study) is associated with higher QOL scores with statistically significant differences in the physical, psychological, and environmental domains. We are considering that having a good salary was significantly and positively related to all QOL domains, and being satisfied at work was positively and significantly associated with the physical, psychological, and environmental dimensions. A stressful job was significantly and negatively associated with the psychological domain. Regarding the health variables, having poor general health is negatively associated with the physical, psychological, and environmental dimensions, while having poor mental health and low social support is significantly and negatively associated with all the domains considered in the study instrument.

For males (Table 1), having a high socioeconomic status is significantly related to a higher score in the environmental dimension, as well as the type of family (>score in people who live alone), carrying out teaching activities and research, earning more than 5 million Colombian pesos (approx. USD 1250), and receiving a salary that allows them to cover the basic needs. Having a salary that allows unforeseen expenses to be covered is positively and significantly associated with the physical, psychological, and environmental domains as well as having a well-paid salary for the work performed. Being satisfied at work is positively and significantly associated with the psychological and environmental domains. Regarding the health variables, practicing sports was positively and significantly related to the psychological dimension, while poor general health was negatively related to the physical domain. Having poor mental health and low social support were significantly and negatively related to all the dimensions considered in the instrument.

#### 3.1.3. Potential Explicative Factors for the Dimensions of the QOL

The multivariate analysis through linear regression (Table 2) showed that the included variables in the models having a negative statistically significant correlation (decrease the scores in QOL) were: poor self-rated health, poor mental health, low social support, stressful job, having a temporary contract, having a lower annual frequency of academic events, and living in places with medium/low socioeconomic status. On the contrary, significant positive significant correlations (increase the QOL scores) were found for the following: having higher working hours per week, working in teaching/research activities, and perceiving that their labor is well-paid. For males, the factors that explain QOL and are negatively related report poor mental health and low social support, while positive correlation was found for a greater annual frequency of academic events, a greater experience as a periodontist in years, and receiving a well-paid salary. The independent variables described in the model explain between 18 and 50% of the QOL scores for women and between 19 and 37% for men (when non-adjusted determination coefficients are considered).

### 3.2. Qualitative Findings as a Key to the Main Quantitative Data

The analysis of the focus groups (FGs) allowed us to explore the factors that were relevant according to the findings that drew attention to the statistical models.

**Figure 1:**
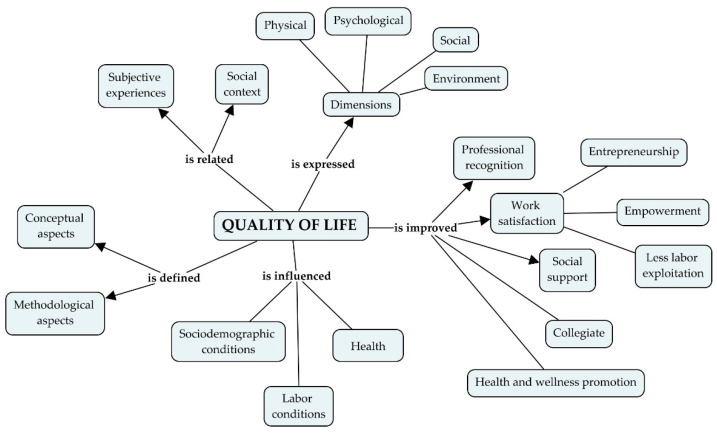
Explicative conceptual map for QOL and its determinants according to the triangulation of findings. Colombia, 2021–2022 (*n* = 187). Elaborated by using Cmaptools 6.03.01.

Figure 1 summarizes the conceptual map to understand the QOL and its determinants. It shows how the QOL is expressed in different dimensions such as physical, psychological, social, and environmental, and is related not only to subjective experiences but is also influenced by the social, economic, and political context. That is why sociodemographic conditions, employment, and the health situation determine and define QOL in conceptual and methodological terms. Finally, it is highlighted that professional recognition, social and labor support, entrepreneurship, and health promotion, among other factors, play a fundamental role in improving the QOL.

The main categories of analysis are shown below (Supplementary Materials Table S3).

#### 3.2.1. Quality of Life: Scope and Definitions of the Concept

“*Quality of life is to be able to be happy with what you do, in what you do, without needing to have many things, living with enough. In my opinion that is quality of life, it’s not about having, but about being and finding happiness in what you do and in your life purpose*”.

(FG 2)

QOL from the periodontists’ perspective did not differ greatly from the theoretical definitions that have been previously established by the scientific literature. They recognized that QOL is an individual concept, which depends on social and individual aspects against a system of standards and values. They understood the importance of happiness and balance in different spheres and spaces (family, work, economics, health, well-being) compared to the fulfillment of a life purpose. Similarly, they considered that being able to reconcile and enjoy family and affective life and free time with their personal and economic support contributes to improving QOL (Supplementary Materials: Table S3, 1a, 1b).

#### 3.2.2. Quality of Life and Working Conditions

“*(…) Working conditions will always be poor for us, not for the owners of the clinics, they always say … I am not the owner of a large clinic because I have experienced work exploitation, that’s the word. They have their point of view as employment creators, as people who have to pay rent, but we are working in totally disadvantageous conditions*”.

(FG 1)

They highlighted the importance of working conditions as a social determinant that impacts well-being and QOL. There are differences while considering the time and work experience of the periodontist. Furthermore, they perceived that newly graduated specialists must work in different workplaces to stabilize economically (Table S3, 2a). However, over time, having a fixed workspace, good marketing and communication strategies, and a strong flow of patients can improve working conditions (Table S3, 2b). Some discourses mentioned the role played by dental clinics that manage personnel in different specialties and patients on a large scale since situations of job insecurity translated into low wages and other adverse working conditions are perceived (Table S3, 2c).

While comparing the working conditions against other specialties, dualities are presented in the perception of the study participants. On the one hand, they stated that some periodontal procedures represent greater economic value when compared to other treatments such as orthodontics, endodontics, and general dentistry. This situation could represent higher income for them (Table S3, 2d). However, on the other hand, they recognized that in some health services, periodontics is less valued when compared to other specialties wherein treatments can be carried out more quickly. These employment and working conditions largely depend on the personal experiences of periodontists in public and private oral health care services (Table S3, 2e). Finally, they mentioned that there are geographical differences between the working conditions of periodontists (Table S3, 2f).

Teaching and research activities are considered as satisfiers of the QOL. Although they do not represent a high economic utility, they possibly generate additional benefits such as interactive activities with the postgraduate students to share academic aspects (Table S3, 2g). Similarly, in line with quantitative data, the participation of periodontists in academic events improves the QOL because it permits social interaction (this interaction was affected in the COVID-19 pandemic because of the mandatory isolation measures). Additionally, these events permit the participants to be at the forefront of dental procedures and materials to improve patient care; in the words of one participant: “feeding our soul” (Table S3, 2h).

#### 3.2.3. QOL and Health

“*Working with the human body will always be an activity that creates high levels of stress, right? What we do is a high-responsibility profession, so anyway we are always subjected to stress and spending long hours sitting in a chair can generate other types of damage, on a physical level, so then you have back pain, leg pain, waist pain, and that will have an effect on your daily life one way or another*”.

(FG 1)

The QOL cannot be delinked from the health concept. From the participants’ perspective, periodontics represents a personal and professional challenge with positive factors. Nevertheless, the academic load and the health care demands for the patients could generate stress, physical wear, and muscular pain, among other symptoms (Table S3, 3a). Resting and experiencing free time were related to QOL and health. Participants’ discourses showed interest in performing daily activities outside the workplace that contribute to generating well-being (Table S3, 3b). They mentioned that the periodontics clinic specialization allows having these spaces (Table S3, 3c).

#### 3.2.4. Proposals for Improving QOL

“*More support among us, more balance in terms of practice and competition among all, not trying to trample on others, but instead enjoying the benefits and the good things of others, and each one working at ease and content with their own things*”.

(FG 2)

Finally, participants in FGs mentioned some situations that could contribute toward improving QOL and are circumscribed in labor and social fields. They ask for greater recognition of the periodontist, higher support and collegiality, training in entrepreneurship in the universities, and less labor exploitation (Table S3, 4a, 4b).

## 4. Discussion

### 4.1. Main Findings and Their Possible Explanations

The major findings of this study showed a QOL reported as high by the periodontists that participated in the study. Differences in scores of the domains of the WHOQOL-BREF were observed, according to sociodemographic, occupational, and health factors. Multivariate analyses showed some factors that have a significant association with QOL in a negative or positive way. Additionally, there were some sex differences in QOL scores. The discourses analyzed confirmed quantitative data, highlighting the influence of the labor conditions in the QOL, and in turn, the relationship that exists between the presence of greater physical demands and stress. In the participants’ opinion, QOL is a concept that is consistent with standards and values and is defined in terms of balance in the spheres of human development that lead to well-being. To our knowledge, this is the first study conducted in Colombia investigating the QOL in periodontists using a mixed-method research design.

This study assumed a gender perspective in data analysis. This is in line with the international set of guidelines to support the systematic presentation of sex and gender in research across all disciplines [24]. The scores of the domains that evaluate QOL show a tendency toward a lower assessment of it within women (with statistically significant differences in the environmental dimension). This aspect differs from the qualitative results wherein the participants’ discourses did not show differences in QOL between both sexes. However, they mentioned that some differences could exist in the self-care practices and the dental care assistance for patients and generate higher stress in women. In the descriptive analyses, some differences between males and females were found: practicing sports (>males), self-rated health (>poor self-rated health in males), and mental health (>poor mental health in females). For instance, a study conducted in Saudi Arabia shows how QOL scores are lower in women (statistically significant differences in the social dimension) [5].

In this regard, some considerations must be taken while interpreting the data. The first aspect to be analyzed is the homogeneity of the sample in sociodemographic, labor, and economic terms. However, specific factors must be analyzed since the multivariate models showed some subtle differences in the perception of the factors that influence QOL in the respondents. For instance, in the physical domain, the QOL is more affected in females by labor and health factors, whereas in males, the QOL is more associated with social support and participation in educative activities. In the social domain, the experience as a periodontist in men was important, and the socioeconomic status was related to the QOL in the environmental dimension for women. All these factors are related to other variables specified in the linear regression interaction models.

The second round of analysis is related to the representations and social constructions made in front of the QOL and other cultural determinants. In this regard, exploring other differential situations between sexes related indirectly to QOL could be interesting. For example, a qualitative study conducted on female dentists in India [25], showed that some aspects could generate unbalance in the workplace conditions for them (less comprehension of specific problems, less empathy, and social support, and some difficulties in conciliating social, labor, and family life). A quantitative study through a cross-sectional survey concludes that women have to balance professional, personal, and social responsibilities [26]. However, the information is limited regarding gender perspective analyses and that is why the results should be cautiously interpreted.

A study conducted in Saudi Arabia showed that when general dentists and specialists are compared, QOL tends to be better for the latter (statistically significant differences in all dimensions) [5]. An Indian study found that having higher educational levels (specialization or master’s degree) is associated with a better QOL (especially in the psychological dimension) [7]. These results are consistent with the qualitative findings; being a periodontist was considered by the focus groups as economically profitable and generating happiness. Furthermore, study participants reported an improvement in their QOL since becoming specialists. Similar aspects were reported by a systematic review wherein greater job satisfaction was found in the specialist dentist [10]. In this sense, factors related to their work and social context, participation in multidisciplinary teams, opportunities for professional development, and labor income may influence QOL.

Complementing the above discussion, QOL was closely related to employment and work conditions, and this was evidenced in the quantitative results wherein a lower score in the environmental domain is related to financial resources, safety, health, and job opportunities in each population [10]. It has also been found that the higher the income of the individual, the higher the level of happiness, and that culture plays an important role in the perception of happiness [27]. In our study, a higher economic income was related to the number of working hours, the experience as a specialist, and having a permanent contract. These factors would benefit the QOL.

Teaching and research activities had a positive influence on QOL (although more in women than in men in the multivariate models). These findings could possibly be explained by the decreasing physical demands and less clinical practice time. These factors prevent the occurrence of physical health disorders [14]. Furthermore, these activities positively influence recognition, concentration, and learning attributes. This is complemented by the results found in the focus groups wherein it was stated that despite the economic remuneration received from teaching, it is lower than the income from clinical practice. The activity is well-valued because they are allowed to share their knowledge, learn new concepts, and stay up to date. A similar study including orthodontic teachers reported multiple reasons to stay in the academic personnel: desire to teach, opportunity to be a mentor for other students, research, advice, and return to the specialty [14].

This study established a strong association between the indicators of self-rated health (especially in women) and mental health (both sexes) with QOL. In the first case, it is important to analyze and consider the possible presence of labor risk factors; for instance, working in uncomfortable positions in the dental office for long periods and assisting a lot of patients during the workday [14,28]. A study conducted in Saudi Arabia found that periodontists were more likely to report musculoskeletal disorders occupational-related [29], and the severity of pain in shoulders was higher in comparison with other clinical dental specialists and general dentists.

This situation is complemented by other studies that mention that scaling and root planning could be the procedures with the highest risk and represent the main cause of musculoskeletal disorders in the hands and arms [30]. An important factor in developing these disorders is the forced pinching that occurs during periodontal therapy [31]. These findings support the need for more studies to identify specific occupational risk factors in periodontists toward preventive measures in health and safety at work.

Poor mental health and labor stress were negatively associated with QOL. Some studies have commented that working in the dental practice is a job that demands both the body and the mind, which would generate chronic stress and harm the psychological health of the professional [5,32,33]; and even more so in contexts of job dissatisfaction or work overload [10,34].This situation was also observed in a similar study conducted on Colombian orthodontists [14]. There are various components of psychological health in the dental profession that affect it, such as requests for time and schedule, work under pressure, lack of appreciation and negative perceptions by patients, hard work to maintain an acceptable lifestyle, and some ethical dilemmas between having more income and the professional ethics, among other factors [5].

Another element of interest is social support, which is related to family, friends, and colleagues’ resources of individuals in their immediate social space. This variable and the participation in academic and social events were considered as positive predictors for QOL in the multivariate analysis for men and women. This relationship was corroborated with focus groups where discourses mentioned that after mandatory social isolation, meeting with colleagues and friends allows individuals to have more interaction and social well-being. In this regard, it is important to mention that there are factors related to greater social control and social support in the work context that contribute to reducing work overload, as mentioned in a study carried out in Sweden [34]. Moreover, the influence that participation in recreational activities, sports, and leisure time has on a better quality of life cannot be excluded [35,36].

### 4.2. Strengths, Weaknesses, and Scope of This Study

While interpreting the results of this study, it is important to consider its strengths and limitations. Data collection instruments were carefully planned and had internal validity processes, both by pilot testing and consistency and validation of the questionnaires related to QOL as well as the reliability and rigor of the qualitative techniques used. The use of mixed models enables a social reality approach through the triangulation of results and their complementarity. As limitations, it is important to consider the circumstances of the sample selection processes, the results cannot be inferred from the general population of specialists, although it is not a population study with probabilistic sampling. The nature of the study does not support causal relationships, but associations between variables and holistic understandings between categories of analysis. Although there was high participation of professionals (*n* = 187), which allowed analysis with a sex/gender perspective, there may be external circumstances that could affect the results of the study since the decision to answer the survey may be related to the QOL perceptions and results could be underestimated. In this regard, a literature review shows that survey response rates among health personnel tend to be lower than the general population due to their demanding work schedules [37].

The effect size analyses using the ETA square revealed values between 0.001 and 0.180 for females and between 0.001 and 0.215 for males (the sociodemographic and labor variables tended to have a small effect size and come health variables had a medium effect size for both sexes). This is consistent with multivariate analyses where variables with greater predictive capacity were those related to health indicators, supplemented with qualitative analyzes. Possible explanations include factors related to sample size and type (relative homogeneity of demographic and work aspects). This same situation concerning the effect of sample size was highlighted in a study of pediatric dentists [17].

Taking into account the scientific literature on the related topic, recognizing the limitations of this study, and the scarcity of studies with multi-method approaches, the findings contribute to the generation of a scientific agenda on this topic of interest. This research can be considered a gateway that, on the one hand, understands the reality of specific groups in the country, and establishes follow-up strategies on issues that concern the clinical dental specialties in social, labor, educational, and QOL aspects. Further research should examine other indicators of quality of life, work, and health.

Finally, the impact of the COVID-19 pandemic on the terms and conditions of Colombian and dental practitioners around the world cannot be ignored [38]. This effect has been found in studies showing the impact on mental health indicators [39], in work well-being [40], and in career plans [41], among other situations. However, the data collection took place during various stages of the social isolation measures proposed by the World Health Organization and the national government. This is an important aspect to analyze because the elements of the questionnaire were not conditioned or were not specific to evaluate the impact of the pandemic. FGs were conducted when social interaction was already permitted, which may influence the results. New studies through qualitative methodologies will make it possible to elucidate the social construction and the factors related to personal and professional expectations after two years from the start of the pandemic.

## 5. Conclusions

In this study, although QOL scores were good for the study participants (the scores surpassed 59 points), multiple factors exert a positive or negative influence. The QOL determinants are explained by subjective aspects (standards and values) including elements of the social and economic context that influence at a personal and professional level. Specifically, in the quantitative component, the factors that had the greatest association on the QOL of periodontists in Colombia were social support, mental and general health, frequency of participation in academic events, work experience as a periodontist, having a well-paid job, stress at work, carrying out teaching/research activities. These associations were corroborated by the discourses in the qualitative component. This situation highlights the particularities of QOL in different social groups, its multifactorial character, and the need for intersectoral and interdisciplinarity for its care, promotion, or research in oral health professionals.

## Figures and Tables

**Table 1:** Differences in QOL scores according to the various sociodemographic, labor, and health variables in the study sample. Medellin, 2021–2022 (*n* = 187).

Variables	WHOQOL-BREF Dimensions															
	Physical				Psychological				Social Relationships				Environment			
	Me	IQR	*p*-Value	Eta Squared	Me	IQR	*p*-Value	Eta Squared	Me	IQR	*p*-Value	Eta Squared	Me	IQR	*p*-Value	Eta Squared
Females																
Sociodemographics																
Marital status																
Married/Cohabitated	67.9	25.0	0.212	0.014	70.8	20.8	0.547	0.001	66.7	25.0	1.000	0.001	59.4	18.8	0.899	0.001
Single/Separated/Widow	73.2	25.0			68.8	29.2			66.7	25.0			59.4	18.8		
Socioeconomic status																
Low/Middle	67.9	21.4	0.264	0.007	66.7	25.0	0.025 *	0.046	58.3	33.3	0.021*	0.040	58.3	15.6	<0.001 ***	0.158
High	73.2	27.7			72.9	24.0			66.7	25.0			70.3	18.8		
Vehicle																
No	62.5	25.0	0.163	0.017	58.3	25.0	0.024 *	0.042	50.0	33.3	0.076	0.029	54.7	14.8	0.007 **	0.065
Yes	71.4	25.0			70.8	20.8			66.7	25.0			62.5	17.2		
Type of family																
Nuclear	71.4	23.2	0.526	0.029	70.8	20.8	0.499	0.031	66.7	25.0	0.323	0.042	59.4	18.8	0.358	0.047
Single-parent	78.6	30.4			75.0	35.4			54.2	47.9			62.5	21.9		
Extended	60.7	---			54.2	---			41.7	---			48.9	---		
Assembled	67.9	33.9			66.7	14.6			66.7	20.8			56.3	31.3		
Live alone	71.4	23.1			75.0	27.1			66.7	22.9			59.4	19.5		
Housing Type																
Own	67.9	25.0	0.486	0.013	66.7	29.2	0.093	0.049	66.7	25.0	0.475	0.018	62.5	18.8	0.080	0.061
Rented	71.4	28.6			62.5	25.0			66.7	33.3			56.3	18.8		
Other	71.4	21.4			75.0	12.5			66.7	25.0			59.4	12.5		
Labor conditions																
Labor activity																
Teaching/Research																
Yes	75.0	25.0	0.191	0.008	72.9	21.9	0.552	0.005	66.7	33.3	0.196	0.018	59.4	25.8	0.340	0.018
No	67.9	25.0			66.7	25.0			66.7	25.0			59.4	15.6		
Clinical assistance																
Yes	71.4	21.4	0.071	0.023	68.8	25.0	0.372	0.010	66.7	25.0	0.619	0.003	59.4	18.8	0.496	0.004
No	---	---			---	---			---	---			---	---		
Administrative																
Yes	60.7	31.3	0.633	0.003	60.4	37.5	0.198	0.031	50.0	56.2	0.229	0.026	62.5	26.6	0.893	0.001
No	71.4	21.4			70.8	20.8			66.7	25.0			59.4	18.8		
Written Contract																
No	67.9	25.0	1.000	0.001	70.8	29.2	0.781	0.001	66.7	33.3	0.409	0.005	62.5	18.8	0.382	0.002
Yes	71.4	22.3			68.8	25.0			66.7	33.3			59.4	16.4		
Several type of contracts																
No	67.9	21.4	0.841	0.001	66.7	27.1	0.609	0.003	66.7	33.3	0.210	0.009	59.4	20.3	0.778	0.003
Yes	71.4	27.7			70.8	20.8			66.7	33.3			59.4	21.9		
Type of employment (by type of contract)																
Independent																
No	71.4	18.8	0.213	0.014	66.7	20.8	0.731	0.002	66.7	41.7	0.359	0.002	59.4	14.1	0.860	0.001
Yes	67.9	25.0			70.8	25.0			66.7	25.0			62.5	21.9		
Provision of services																
No	71.4	22.3	0.248	0.011	70.8	29.2	0.489	0.002	66.7	33.3	0.105	0.021	62.5	20.3	0.311	0.007
Yes	67.9	25.0			66.7	25.0			66.7	33.3			59.4	15.6		
Percentage rent																
No	69.6	23.2	0.906	0.001	70.8	25.0	0.073	0.023	66.7	27.1	0.643	0.001	62.5	18.8	0.002 **	0.076
Yes	71.4	21.4			62.5	20.8			66.7	33.3			56.3	15.6		
Temporary																
No	71.4	22.3	0.666	0.003	70.8	22.9	0.407	0.005	66.7	25.0	1.000	0.001	60.9	18.8	0.216	0.019
Yes	71.4	25.0			66.7	20.8			66.7	25.0			59.4	9.4		
Permanent																
No	67.3	22.3	0.019 *	0.038	66.7	25.0	0.032*	0.040	66.7	25.0	0.538	0.003	59.4	17.2	0.009 **	0.070
Yes	82.1	14.3			79.2	16.7			75.0	41.7			75.0	21.9		
Monthly income (Colombian peso)																
< 5,000,000	64.3	21.4	0.001 **	0.086	66.7	25.0	0.023 *	0.048	66.7	33.3	0.056	0.030	59.4	12.5	<0.001 ***	0.128
≥ 5,000,001	76.8	17.9			70.8	26			70.8	27.1			67.2	21.9		
Does your current salary allow you to cover your basic needs, and those of the people who depend on you?																
No	67.9	28.6	0.685	0.001	68.8	17.7	0.490	0.003	54.2	35.4	0.291	0.012	59.4	10.2	0.325	0.014
Yes	71.4	21.4			70.8	25.0			66.7	25.0			59.4	18.8		
Does your current salary allow you to cover unforeseen important expenses?																
No	64.3	20.5	0.031 *	0.040	66.7	20.8	0.052	0.031	66.7	31.3	0.425	0.007	56.3	12.5	<0.001 ***	0.119
Yes	71.4	23.2			70.8	25.0			66.7	25.0			62.5	18.8		
Do you think you are well-paid for the work you do and the time you dedicate to it?																
No	64.3	24.1	0.002 **	0.077	66.7	24.0	0.003 **	0.086	66.7	33.3	0.009**	0.069	56.3	15.6	<0.001 ***	0.153
Yes	75.0	17.9			70.8	25.0			75.0	25.0			65.6	18.8		
Labor satisfaction																
Satisfied	75.0	21.4	0.013 *	0.046	70.8	25.0	0.047*	0.031	66.7	25	0.321	0.014	62.5	18.8	0.001 **	0.102
Unsatisfied	66.1	18.8			64.6	20.8			62.5	33.3			56.3	13.3		
Level of stress at work																
Not stressful	78.6	21.4	0.051	0.029	83.3	29.2	0.049	0.034	66.7	33.3	0.891	0.001	65.6	15.6	0.050	0.019
Stressful	69.6	25.0			66.7	25.0			66.7	25.0			59.4	16.4		
Health																
Sport practice																
Yes	71.4	25.0	0.206	0.008	70.8	20.8	0.353	0.009	66.7	25.0	0.452	0.006	62.5	18.8	0.058	0.026
No	67.9	23.2			66.7	25.0			58.3	33.3			59.4	15.6		
Self-rated health																
Good	71.4	21.4	<0.001 ***	0.180	70.8	20.8	0.001 **	0.121	66.7	25.0	0.541	0.006	59.4	15.6	0.032*	0.036
Poor	53.6	21.4			54.2	22.9			66.7	33.3			50	25		
Mental health (GHQ-12)																
Good	78.6	10.7	<0.001 ***	0.118	75	20.8	<0.001 ***	0.167	75	25.0	0.004 **	0.067	62.5	15.6	0.028 *	0.043
Poor	64.3	22.3			62.5	20.8			66.7	33.3			59.4	22.7		
Social support (Duke-UNC-11)																
Normal	71.4	21.4	<0.001***	0.122	70.8	25.0	0.001 **	0.110	66.7	27.1	<0.001 ***	0.147	59.4	17.2	0.007**	0.073
Low	53.6	17.9			58.3	20.8			41.7	16.7			53.1	21.9		
Males																
Sociodemographics																
Marital status																
Married/Cohabitated	71.4	14.3	0.120	0.043	75.0	20.8	0.547	0.011	66.7	25.0	0.925	0.002	65.5	21.9	0.288	0.033
Single/Separated/Widow	75.0	14.3			75.0	25.0			66.7	25.0			68.8	28.1		
Socioeconomic status																
Low/Middle	71.4	19.6	0.232	0.028	70.8	17.7	0.145	0.041	66.7	25.0	0.061	0.037	62.5	23.4	0.005 **	0.108
High	75.0	14.3			75.0	20.8			75.0	25.0			71.9	15.6		
Vehicle																
No	71.4	21.4	0.781	0.002	79.2	14.6	0.263	0.004	66.7	29.2	0.656	0.001	65.6	9.4	0.943	0.001
Yes	75.0	16.1			70.8	20.8			66.7	25.0			68.8	25.0		
Type of family																
Nuclear	71.4	14.3	0.096	0.068	75.0	20.8	0.082	0.088	66.7	20.8	0.312	0.102	65.6	20.3	0.015 *	0.148
Single-parent	60.7	---			52.1	---			33.3	---			48.9	---		
Live alone	78.6	18.8			79.2	27.1			70.8	27.1			73.4	29.7		
Housing Type																
Own	75.0	14.3	0.949	0.009	70.8	20.8	0.914	0.002	66.7	25.0	0.476	0.024	65.6	25.0	0.734	0.061
Rented	73.2	22.3			75.0	12.5			58.3	33.3			67.2	20.3		
Other	71.4	39.3			79.2	29.2			70.8	25.0			71.9	39.8		
Labor conditions																
Labor activity																
Teaching/Research																
Yes	78.6	19.6	0.172	0.030	75.0	18.8	0.265	0.029	75.0	20.8	0.081	0.045	75.0	25.0	0.030 *	0.066
No	71.4	16.1			70.8	20.8			66.7	16.7			62.3	18.8		
Clinical assistance																
Yes	75.0		0.856	0.010	75.0	20.6	0.735	0.035	66.7	22.9	1.000	0.001	67.5	24.2	0.782	0.026
No	64.3	---			58.3	---			66.7	---			51.6	---		
Administrative																
Yes	73.2	---	0.960	0.001	79.2	---	0.481	0.006	54.2	---	0.501	0.013	67.2	---	0.986	0.001
No	75.0	17.0			75.0	20.8			66.7	25.0			67.2	25.0		
Written Contract																
No	75.0	25.9	0.849	0.010	72.9	22.9	0.549	0.013	66.7	27.1	0.887	0.003	65.6	29.7	0.479	0.010
Yes	73.2	14.3			75.0	16.7			66.7	16.7			68.8	24.2		
Several type of contracts																
No	73.2	23.2	0.484	0.022	75.0	20.8	0.923	0.006	66.7	20.8	0.909	0.005	65.6	24.2	0.261	0.024
Yes	75.0	14.3			72.9	20.8			66.7	25.0			68.8	25.0		
*Type of employment (by contract)*																
Independent																
No	75.0	15.2	0.909	0.001	66.7	27.1	0.207	0.015	66.7	27.1	0.986	0.001	65.6	22.7	0.759	0.001
Yes	71.4	17.0			75.0	16.7			66.7	16.7			68.8	25.0		
Provision of services																
No	71.4	14.3	0.905	0.003	75.0	20.8	0.415	0.015	66.7	25.0	0.241	0.033	62.6	21.9	0.824	0.002
Yes	75.0	17.9			75.0	16.7			66.7	25.0			68.8	25.0		
Percentage rent																
No	75.0	15.2	0.414	0.010	75.0	21.9	0.391	0.009	66.7	25.0	0.059	0.049	68.8	25.0	0.099	0.025
Yes	71.4	19.6			70.8	19.8			58.3	25.0			64.1	23.4		
Temporary																
No	75.0	17.0	0.930	0.001	75.0	20.8	0.874	0.001	66.7	25.0	0.874	0.001	65.6	24.2	0.645	0.004
Yes	73.2	20.5			75.0	10.4			66.7	18.8			70.3	27.3		
Permanent																
No	73.2	17.9	0.431	0.008	75.0	20.8	0.971	0.001	66.7	18.8	0.538	0.002	65.6	22.7	0.245	0.018
Yes	75	18.8			77.1	19.8			62.5	33.3			68.8	15.6		
Monthly income (Colombian peso)																
<5,000,000	71.4	25.0	0.113	0.067	75.0	29.2	0.271	0.040	66.7	16.7	0.721	0.003	56.3	18.8	0.001 **	0.161
≥5,000,001	75.0	14.3			75.0	20.8			66.7	25.0			68.8	18.8		
Does your current salary allow you to cover your basic needs, and those of the people who depend on you?																
No	62.5	41.1	0.105	0.018	62.5	40.6	0.149	0.077	58.3	39.6	0.357	0.025	45.3	23.4	0.002 **	0.163
Yes	75.0	14.3			75.0	20.8			66.7	25.0			68.8	24.2		
Does your current salary allow you to cover unforeseen important expenses?																
No	60.7	21.4	0.004 **	0.147	54.2	25.0	0.006 **	0.150	58.3	16.7	0.064	0.043	53.1	21.9	<0.001 ***	0.174
Yes	75.0	14.3			75.0	16.7			66.7	25.0			68.8	21.9		
Do you think you are well-paid for the work you do and the time you dedicate to it?																
No	67.9	19.6	0.001 **	0.154	70.8	16.7	0.054	0.067	66.7	20.8	0.077	0.029	59.4	17.2	<0.001 ***	0.215
Yes	75.0	16.1			75.0	20.8			75.0	25.0			75.0	18.8		
Labor satisfaction																
Satisfied	75.0	14.3	0.069	0.059	75.0	18.8	0.031*	0.095	66.7	25.0	0.222	0.013	68.8	23.4	0.003 **	0.142
Unsatisfied	67.9	17.9			66.7	25.0			66.7	16.7			56.3	25.0		
Level of stress at work																
Not stressful	80.4	18.8	0.139	0.022	77.1	19.8	0.279	0.017	75.0	25.0	0.076	0.024	68.8	30.5	0.735	0.002
Stressful	71.4	15.2			75.0	20.8			66.7	20.8			65.6	25.0		
Health																
Sport practice																
Yes	75.0	14.3	0.051	0.058	75.0	25.0	0.014*	0.112	66.7	25.0	0.445	0.003	65.6	21.9	0.103	0.049
No	67.9	14.3			66.7	20.8			66.7	25.0			68.8	25		
Self-rated health																
Good	75.0	14.3	0.004 **	0.165	75.0	20.8	0.401	0.032	66.7	25.0	0.084	0.014	67.2	25	0.299	0.029
Poor	57.1	29.5			70.8	34.4			54.2	14.6			62.5	33.6		
Mental health (GHQ-12)																
Good	78.6	10.7	0.047 *	0.054	79.2	15.6	0.001 **	0.137	75	22.9	0.014 *	0.083	68.8	15.6	0.041 *	0.061
Poor	71.4	18.8			66.7	14.6			66.7	25.0			62.6	23.4		
Social support (Duke-UNC-11)																
Normal	75.0	14.3	<0.001 ***	0.225	75.0	17.7	0.007 **	0.108	70.8	25.0	0.004 **	0.144	68.8	25.0	0.015 *	0.069
Low	57.1	13.4			54.2	22.9			54.2	35.4			51.6	21.9		

Mann–Whitney U test for dichotomous variables, Kruskal–Wallis test for polychotomous variables. IQR: Interquartile range. * *p*-value < 0.05; ** *p*-value < 0.01; *** *p*-value < 0.001.

**Table 2:** Lineal regression models for the scores of the WHOQOL-BREF dimensions of QOL according to the variables included in the study sample. Medellín, 2021–2022 (*n* = 187).

WHOQOL-BREF Dimensions	Variables Included in the Lineal Regression Model	Determination Coefficient (%R2)		Change of R2%	*p*-Value Change of R2%	Non-Standardized Regression Coefficient	Standardized Regression Coefficient	*p*-Value	F-Value	*p*-Value (Model)	Durbin-Watson Statistic
		Unadjusted	Adjusted								
**Females**											
Physical	Working hours per week	50.0	45.0	3.4	0.032	0.304	0.095 **	0.002	9.934	<0.001	1.879
	Self-rated health (poor)					−12.643	3.455 ***	<0.001			
	Mental health (poor)					−7.670	2.737 **	0.007			
	Social support (Low)					−10.766	3.818 **	0.006			
	Level of stress at work (Stressful)					−8.243	3.389 *	0.018			
	Labor activity: Teaching/researcher					12.109	3.923 **	0.003			
	Temporary contract					−9.260	4.327 *	0.032			
Psychological	Mental health (poor)	26.0	24.0	7.4	0.007	−12.219	2.818 ***	<0.001	13.301	<0.001	2.119
	Self-rated health (poor)					−10.274	3.723 ***	<0.001			
Social relationships	Social support (Low)	18.0	16.0	4.8	0.038	−25.932	6.829 ***	<0.001	8.385	0.001	1.930
	Annual frequency of participation in events of training and unformal education					−2.015	0.954 *	0.038			
Environment	Socioeconomic status (Low-Middle)	19.0	16.0	6.2	0.019	−8.809	2.716 **	0.002	8.651	<0.001	1.946
	Do you think you are well-paid for the work you do and the time you dedicate to it? (Yes)					6.836	2.851 *	0.019			
**Males**											
Physical	Social support (Low)	36.0	33.0	8.9	0.009	−23.270	4.476 ***	<0.001	14.669	<0.001	2.458
	Annual frequency of participation in events of training and unformal education					1.821	0.671 **	0.009			
Psychological	Social support (Low)	34.0	30.0	7.0	0.021	−17.916	4.493 ***	<0.001	8.917	<0.001	1.826
	Mental health (poor)					−9.268	3.190 **	0.005			
	Annual frequency of participation in events of training and unformal education					1.599	0.674 *	0.021			
Social	Social support (Low)	37.0	33.0	8.6	0.010	−24.089	5.953 ***	<0.001	10.170	<0.001	1.865
	Mental health (poor)					−13.096	4.296 **	0.004			
	Periodontist experience (years)					0.744	0.279 *	0.01			
Environment	Do you think you are well-paid for the work you do and the time you dedicate to it? (Yes)	23.0	22.0	22.9	<0.001	15.106	3.768 ***	<0.001	16.071	<0.001	2.327

* *p*-value < 0.05; ** *p*-value < 0.01; *** *p*-value < 0.001. Method for the multivariate lineal regression: Stepwise.

## Data Availability

Quantitative data presented in this study are available upon reasonable request from the corresponding author.

## Supplementary Materials

The following supporting information can be downloaded at: https://www.mdpi.com/article/10.3390/ijerph192316102/s1. Table S1: Sociodemographic, labor, quality of life, and health characteristics of the study population. Colombia, 2021–2022 (*n* = 187). Table S2: Bivariate correlations between the WHOQOL-BREF scores of QOL and the sociodemographic and labor variables in the study sample. Medellin, 2021–2022 (*n* = 187). Table S3: Verbatim quotes from the perceptions of the participants in the FGs (*n* = 2) about QOL and QOL determinants. Colombia, 2021–2022.
