# Supplementary material for: A Comprehensive Mixed Methods Approach for Studying the Quality of Life of Colombian Periodontists
Source: Int J Environ Res Public Health. 2022 Dec 1;19(23):16102. doi: 10.3390/ijerph192316102 (PMC9736974; doi:10.3390/ijerph192316102)
Supplement: Supplementary file 1 [file ijerph-19-16102-s001.zip › ijerph-2029866-supplementary.pdf]

Table S1. Sociodemographic, labor, quality of life and health characteristics of the study population. Colombia, 2021-2022 (n=187).

Variables	Females		Males	
	n	%	n	%
**Sociodemographics**				
Age[a]				
(Me, IQR)	40.0	13.0	43.0	13.0
Marital Status				
Single	31	27.4	16	21.6
Married/Cohabitated	73	64.6	55	74.3
Separate	7	6.2	3	4.1
Widow	2	1.8	0	0.0
Socioeconomic status				
Low	2	1.8	2	2.7
Middle	59	52.2	36	48.6
High	52	46.0	36	48.6
Vehicle				
No	20	17.7	13	17.6
Yes	93	82.3	61	82.4
Type of family				
Nuclear	77	68.1	57	77.0
Single-parent	10	8.8	2	2.7
Extended	3	2.7	1	1.4
Assembled	9	8.0	0	0.0
Live alone	14	12.4	14	18.9
Housing Type				
Own	71	62.8	50	67.6
Rented	27	23.9	18	24.3
Other	15	13.3	6	8.1
**Labor conditions**				
DDS Experience (years)[a]				
(Me, IQR)	17.0	13.0	19.5	9.5
Periodontist experience (years)[a]				
(Me, IQR)	10	10.0	9.0	8.0
*Labor activity (Yes)**				
Teaching/Research	34	30.1	21	28.4
Clinical assistance	112	99.1	72	97.3
Administrative	8	7.1	2	2.7
Written Contract				
No	47	41.6	34	45.9
Yes	66	58.4	40	54.1
Several type of contracts				
No	49	43.4	26	35.1
Yes	64	56.6	48	64.9
Type of contract[b]				

Variables	Females		Males	
	n	%	n	%
Independent	71	62.8	48	64.9
Provision of services	71	62.8	39	52.7
Percentage rent	39	34.5	24	32.4
Temporary	19	16.8	10	13.5
Permanent	11	9.7	12	16.2
Percentage rent value[a]				
(Me, IQR)	50.0	10.0	60.0	10.0
Working hours per week[a]				
(Me, IQR)	35.0	20.0	40.0	15.0
Resting days per week[a]				
(Me, IQR)	2.0	1.0	2.0	1.0
Monthly income (Colombian peso)[c]				
< 3,000,000	18	15.9	4	5.4
3,000,001- 4,000,000	28	24.8	7	9.5
4,000,001- 5,000,000	17	15.0	8	10.8
5,000,001- 6,000,000	29	25.7	12	16.2
≥ 6,000,001	21	18.6	43	58.1
Does your current salary allow you to cover your basic needs, and those of the people who depend on you?				
No	10	8.8	6	8.1
Yes	103	91.2	68	91.9
Does your current salary allow you to cover unforeseen important expenses?				
No	32	28.3	11	14.9
Yes	81	71.7	63	85.1
Do you think you are well-paid for the work you do and the time you dedicate to it?				
No	68	60.2	33	44.6
Yes	45	39.8	41	55.4
Labor satisfaction				
Satisfied	79	69.9	61	82.4
Unsatisfied	34	30.1	13	17.6
Level of stress at work				
Not stressful	23	20.4	12	16.2
Stressful	90	79.6	62	83.8
Annual frequency of participation in events of training and unformal education				
(Me, IQR)	2.0	1.0	2.0	2.0
**Health**				
Sport practice				
Yes	64	43.4	55	74.3
No	49	56.6	19	25.7

Variables	Females		Males	
	**n**	**%**	**n**	**%**
Self-rated health				
Good	96	85.0	66	89.2
Poor	17	15.0	8	10.8
Mental health (GHQ-12)				
Good	43	38.1	28	37.8
Poor	70	61.9	46	62.2
Social support (Duke-UNC-11)				
Normal	98	86.7	66	89.2
Low	15	13.3	8	10.8
**Quality of Life**				
Physical[a]				
(Me, IQR)	71.4	21.4	75.0	15.2
Psychological[a]				
(Me, IQR)	70.8	25.0	75.0	20.8
Social[a]				
(Me, IQR)	66.7	25.0	66.7	25.0
Environment[a]				
(Me, IQR)	59.4	18.8	67.2	25.0
**All**	**113**	**60.4**	**74**	**39.6**

[a] Kolmogorov-Smirnov test for Normality. Skewness and kurtosis values for the domains of the WHOQOL-BREF domains are: physical (-0.403; -0.403); psychological (-0.349; -0.243); social relationships (-0.532; 0.074), environment (0.054; 0.017). For the other study variables, the values (including both sexes) are: Age (0.514; 0.040); years of experience as a dentist (0.610; 0.272); years of experience as a specialist (1.245; 1.493); percentage rent value (-0.114; 1.187); working hours per week (-0.296; -0.189); resting days per week (0.995; 1.255). [b] Non-mutually exclusive percentages. [c] 1 U$ Dollar = 4,000 pesos (at the time of the study). IQR: Interquartile range.

Table S2. Bivariate correlations between the WHOQOL-BREF scores of QOL and the sociodemographic and labor variables in the study sample. Medellin, 2021-2022 (n= 187).

Variables	WHOQOL-BREF dimensions			
	Physical	Psychological	Social	Environment
**Females**				
Age	-0.037	0.078	0.006	0.189*
DDS Experience (years)	-0.021	0.115	0.023	0.242*
Periodontist experience (years)	-0.025	0.101	-0.009	0.229*
Percentage rent value	0.043	-0.041	-0.098	-0.003
Working hours per week	0.364***	0.144	0.174	0.084
Resting days per week	-0.137	-0.042	-0.094	0.04
Annual frequency of participation in events of training and unformal education	0.016	0.02	-0.087	0.032
**Males**				
Age	0.015	0.035	0.212	0.071
DDS Experience (years)	0.029	0.024	0.206	0.104
Periodontist experience (years)	-0.025	0.077	0.300**	0.161
Percentage rent value	0.105	0.084	0.017	0.150
Working hours per week	0.131	0.281*	-0.007	0.22
Resting days per week	0.152	-0.016	-0.142	0.143
Annual frequency of participation in events of training and unformal education	0.171	0.122	0.156	0.260*

Spearman's rank correlation coefficient. * p-value < 0.05; ** p-value < 0.01; *** p-value <0.001.

Table S3. Verbatim quotes from the perceptions of the participants in the FGs (n= 2) about QOL and QOL determinants. Colombia, 2021-2022.

Categories	Keywords	Excerpts from participants' discourses
**1) Quality of life: scope and definitions of the concept**	Quality of life, standards, and values	*a) "It is a set of indicators that assess the well-being of each person, but this goes alongside the individual's social and the psychological aspect and well, it involves more things that would play a role when evaluating what is quality of life. For example, the social aspect, where the person lives, the financial situation, I don't know about their health, if they live in the city or in the countryside... the quality of life is determined by many factors in their surroundings" (FG 1)*
	Quality of life and balance	*b) "(..) I have like a really balanced life, I have time to be a mom, a wife, a friend, I do a lot of things, [laughs] I think it has mostly to do with each person's amount of energy and how they distribute time, I don't think periodontics prevents you from experiencing other spaces, not at all" (FG 2)*
**2) Quality of life and working conditions**	Multiple workplaces	*a) "(...) I graduated six years ago from the postgraduate course in periodontology. At the beginning I was working in some clinics for a percentage of the earnings, but soon, in less than 2 years I quit doing that, and I focused mainly in having my own practice"(FG 1).*
	Job stability in private practices	*b) "(...) I don't work for less than 60% anywhere. I set that as a goal, and I have tried to maintain it. I have been offered jobs that I can say are very good, but the payment is quite lower, so I prefer to say no and keep working in the consultations, which I have been doing for five years already" (FG 1)*
	Social networks and media	*c) "Today I see young colleagues using social media a lot. They promote themselves a lot in Instagram and Facebook, that type of thing, and I see that some of them do very well, others not so much, some manage to build a reputation within the groups of patients the work with. It changes when they see high or middle- and lower-class patients and yet I have managed to meet and there are some students I've had contact with, that I see that have managed to handle this type of promotions very well in the middle and lower classes, and they do quite well" (FG 2)*
	Periodontal treatments costs	*d) "In my experience, it's more profitable to be a periodontist, we work, we work less hours that an orthodontist does, sometimes an orthodontist has to see 60 patients to earn as much as a periodontist can make doing two implant surgeries" (FG 1)*
	Low appreciation of periodontics	*e) "There are clinics that at first don't hire a periodontist and because they don't know the profession, they believe that it is not necessary. I have worked in several clinics, fortunately I already established myself in one, but there are many where the periodontist is not as very taken into account: orthodontics is worth more, endodontics is worth more, rehabilitation is worth more, because they are things that get done faster, so they make money within orthodontics, but they fail to realize that periodontics is an area that can produce very good money when it's well managed" (FG 2)*
	Geographic differences in work	*f) "I think it also depends a lot on the geographical location, there are cities where perhaps people have more purchasing power, in small cities the purchasing power is lower. Let's say that in cities like Pereira, Armenia, Manizales they have less purchasing power than in cities like Bogotá or Medellín" (FG 2)*

	Teaching as a satisfaction factor	*g) "My experience as a teacher has been a very rewarding experience, teaching is never rewarding from the economic perspective, it is a rewarding experience because you share with people, because you have the possibility of sharing what you have built and what you have learned through your professional practice" (Focus Group 2)*
	Participation in academic events	*h) "Continuing education in congresses strengthens our knowledge, we reinforce the knowledge we have, we feed ourselves with new knowledge and I also totally agree that it feeds our soul, meeting with all our colleagues, with our friends, with those we had not seen in a long time. Sharing is very important, and I think that now that the pandemic is ending and we are able to return to those spaces, those scenarios, it's wonderful again." (FG 2)*
**3) Quality of life and health**		*a) "I think periodontics is a very nice profession, we have a lot of things that I personally love, to create and to do something nice for the patient. I think that part is very satisfying, but sometimes there are moments of stress. I work in an institutional context and in an institutional context it's sometimes difficult to reject or choose patients, so there are times when there's indeed stress, particularly caused by the behavior of patients, because they are difficult to handle and so these are moments that do create difficult situations, that you eventually, with the experience, learn how to handle and to work with" (FG 2)*
	Rest and leisure time	*b) "I like to read, go to the movies, I like to have lunch and the lunch to be followed by an afternoon with wine, I like to study, I like to make bread, I like to cook, I like to talk with my husband, go for a walk, jog too, that is, anything that comes up and catches my attention, I will be there because life is a just a short moment" (FG 1)*
		c) "I do have free time left. In my practice, as I told you before, I value time very much and something I have learned is to say no, that is, to say I work up to this point and my time is very valuable. So yes, I have a lot of free time. I try to share it with friends, particularly I have no family, I am not married, so with friends, I really like to go cycling, go for a walk, I don't know. So yes, thank God periodontics allows me the space to have free time (FG 2)
**4) Proposals to improve quality of life**	Professional appreciation	*a) "We have to make our profession be valued, both by patients and other colleagues, as well as by all those who need us to value what we do. Perhaps we can change the idea that, for example, someone that just graduated goes out to earn a small percentage in some clinics and so on... perhaps specially on that aspect to try to improve job opportunities for people who are just graduating from a specialty, creating a sense of value of the profession among ourselves and among our colleagues" (FG 2)*
	Entrepreneurship and business training	*b) "Graduates of XXX (name omitted for confidentiality) lack a little more training as entrepreneurs because we are set to think that we are going to work for someone else, for this clinic. I think that our mentality has to change a little and we go out to start companies, practices, to be the owners of clinics. That is definitely going to make an impact" (FG 1)*
